# Pyrethroids and Nectar Toxins Have Subtle Effects on the Motor Function, Grooming and Wing Fanning Behaviour of Honeybees (*Apis mellifera*)

**DOI:** 10.1371/journal.pone.0133733

**Published:** 2015-08-17

**Authors:** Caitlin J. Oliver, Samantha Softley, Sally M. Williamson, Philip C. Stevenson, Geraldine A. Wright

**Affiliations:** 1 Centre for Behaviour and Evolution, Institute of Neuroscience, Newcastle University, Newcastle upon Tyne, United Kingdom; 2 School of Biology, Newcastle University, Newcastle upon Tyne, United Kingdom; 3 Jodrell Laboratory, Royal Botanic Gardens, Kew, Surrey, United Kingdom; 4 Natural Resources Institute, University of Greenwich, Chatham, United Kingdom; University of Guelph, CANADA

## Abstract

Sodium channels, found ubiquitously in animal muscle cells and neurons, are one of the main target sites of many naturally-occurring, insecticidal plant compounds and agricultural pesticides. Pyrethroids, derived from compounds found only in the Asteraceae, are particularly toxic to insects and have been successfully used as pesticides including on flowering crops that are visited by pollinators. Pyrethrins, from which they were derived, occur naturally in the nectar of some flowering plant species. We know relatively little about how such compounds—i.e., compounds that target sodium channels—influence pollinators at low or sub-lethal doses. Here, we exposed individual adult forager honeybees to several compounds that bind to sodium channels to identify whether these compounds affect motor function. Using an assay previously developed to identify the effect of drugs and toxins on individual bees, we investigated how acute exposure to 10 ng doses (1 ppm) of the pyrethroid insecticides (cyfluthrin, tau-fluvalinate, allethrin and permethrin) and the nectar toxins (aconitine and grayanotoxin I) affected honeybee locomotion, grooming and wing fanning behaviour. Bees exposed to these compounds spent more time upside down and fanning their wings. They also had longer bouts of standing still. Bees exposed to the nectar toxin, aconitine, and the pyrethroid, allethrin, also spent less time grooming their antennae. We also found that the concentration of the nectar toxin, grayanotoxin I (GTX), fed to bees affected the time spent upside down (i.e., failure to perform the righting reflex). Our data show that low doses of pyrethroids and other nectar toxins that target sodium channels mainly influence motor function through their effect on the righting reflex of adult worker honeybees.

## Introduction

Honeybees (*Apis mellifera*) are among the most economically valuable animal pollinators on which one third of worldwide crop productivity depends [1, 2]. The annual economic value of honeybees, as pollinators alone, is estimated to be £190 million in the United Kingdom [3] and $0.15–19 billion per year in the United States of America [4]. However, the number of honeybee colonies has declined in recent years in many countries [5, 6]. Understanding why these declines are occurring is essential, if we are to ensure pollinator prosperity and food security. Evidence suggests that declines are attributable to an interactive effect of parasites, pathogens, agricultural intensification and pesticide usage [7–9], but pesticides could play a critical role [10].

Most research to date on how pesticides influence bees has focused on neonicotinoids. Sub-lethal doses of these pesticides have an adverse effect on honeybee navigation [11–13], learning and memory [14] and motor function [15, 16]. For these reasons, three of the most commonly used neonicotinoids, clothianidin, imidacloprid and thiamethoxam, were restricted for use in the European Union for two years [17]. Opponents of this neonicotinoid moratorium argue that pesticides, with potentially worse consequences for pollinators, will be used in their place. Among these pesticides are pyrethroids [7]. Synthetic pyrethroids are pesticides that are derived from one of six natural pyrethrins, cinerin I, of the pyrethrum flower, *Tanacetum cinerariifolium* [18]. These compounds target S6 segments of voltage gated sodium channels found in insect nervous systems [19]. Their main mode of action as insecticides is to cause paralysis [19], and they have relatively higher LD_50_ values for insects compared to neonicotinoids (Table 1). Pyrethroids may have similar sub-lethal effects to neonicotinoids on bee health but fewer studies have investigated this [7, 20].

**Table 1:** LD_50_ Values and Concentrations of Compounds. LD_50_ values of pyrethroids [5, 21–23] and their maximum detected concentrations in wax, pollen and bees as well as the total detected concentration [5]. The mean LD_50_ value was calculated and displayed when several LD_50_ values had been reported in the literature.

Pyrethroids	Dose (ng/bee)	LD_50_ (ng/μl)	LD_50_ (ppm)	Wax (ppm)	Pollen (ppm)	Bees (ppm)	Total (ppm)
Cyfluthrin	10	0.22	0.22	0.045	0.034	0	0.079
Tau-Fluvalinate	10	1.56	1.56	204	2.67	5.86	213
Permethrin	10	0.705	0.705	0.372	0.092	19.6	20
Allethrin	10	48.8	48.8	1.1	0.1	0.4	1.6

Bees and other pollinators encounter toxins such as pesticides and naturally-occurring plant toxins in the nectar and pollen of plants on which they feed [24–26]. Nectar toxins include sodium channel activators, although there are no reports of naturally occurring pyrethrins or synthetically-produced pyrethroids in nectar to date. For example, the aconitine-like compounds, lappaconitine, leucostine A and 6-O-acetylacosepticine, are found in the nectar of monkshood species (*Aconitum septentrionale)* [25]. Pyrethrins have been found in floral tissues including seeds [27] and so it is possible that they occur naturally, but this has not been previously reported.

As well as to naturally occurring secondary metabolites, honeybees may be exposed to widely used synthetic pyrethroids whilst foraging [20]. Choudhary and Sharma [28] found that the pyrethroid, λ-cyhalothrin remained in nectar and pollen of the mustard plant (*Brassica juncea*) on which honeybees forage, for 72 h post application. Furthermore, at the point of application, the concentration of λ-cyhalothrin was 0.79 ppm in nectar and 1.52 ppm in the pollen of *B*. *juncea*. Bees also have the potential to be exposed to pyrethroids when they feed on aphid honeydew, because many aphid species have become resistant to pyrethroid insecticides and excrete these compounds in honeydew [29–31].

The likelihood of bees encountering high doses of pyrethrins/pyrethroids and nectar toxins in the field varies depending on the compound. For example, bees may encounter concentrations of up to 7.5 ppm of tau-fluvalinate within the hive, because this compound is deliberately administered by beekeepers as an in-hive miticide [5, 32, 33]. Mullin et al. [5] also found permethrin, at concentrations of 2.5 ppm, in honeybee hives. However, the same study only detected cyfluthrin, at concentrations of 0.01 ppm. GTX, pyrethrin and aconitine have been reported to be present in nectar at concentrations of ~50 ppm [26], 1 ppm [34] and ~1 ppm [25], respectively.

**Fig 1:**
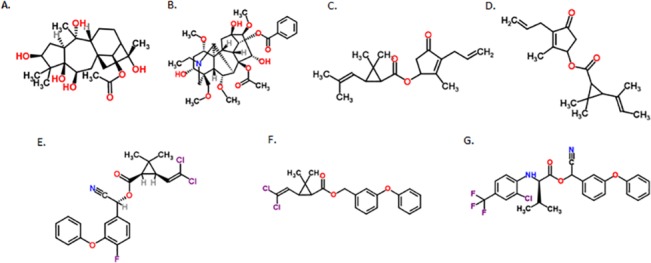
Chemical structures of compounds used in this study. (A) grayanotoxin I [38]; (B) aconitine [39]; (C) pyrethrin [40]; (D) allethrin [41]; (E) cyfluthrin [21]; (F) permethrin [22]; (G) tau-fluvalinate [23].

Previous studies have investigated the effects of pyrethroids on survival and learning and memory in bees [3, 5–7, 20, 35] but none have investigated their influence on honeybee motor function. Assays of motor function can often reveal subtle effects on behaviour that are not revealed in survival studies. Here, we used an assay of motor function, used previously to assess how drugs and toxins affect honeybee behaviour [15, 16, 36, 37]. We used four synthetic pesticides, permethrin, cyfluthrin, allethrin, and tau-fluvalinate (Fig 1). All of these compounds are used either as pesticides on crops or as miticide treatments within honeybee colonies. We also tested two nectar toxins known to influence sodium channels: aconitine and GTX. These compounds bind to different sites on voltage gated sodium channels.

## Materials and Methods

### Honeybees

Honeybees (*Apis mellifera* var. Buckfast) were obtained from the National Bee Unit, York, UK. Honeybees were maintained outdoors between June and August 2013 at Newcastle University. They were allowed to forage freely and had not been treated with chemical mite treatments with oxalic acid for approximately six months before experimentation. Forager bees were collected from outside the hive, every afternoon, in small plastic vials, cold anaesthetised for 3–4 min, and then restrained in a brass harness as in [42]. Each bee was fed 1 M sucrose to satiety and then placed in a humidified plastic box overnight. Approximately 18–24 h after harnessing, bees were fed 10 μl of a treatment solution using a 2-ml Gilmont micrometer syringe (GS-1200). Approximately 45 min after feeding, bees were transferred to a 150 x 15 mm Petri dish, where they were left to acclimatise for 15 min. Each bee was observed for 10 min continuously. For the pyrethroid/nectar toxin study, the behaviour of 16 bees was analysed per treatment (n = 6 x 16 = 96 bees in total). For the GTX dose dependent study, the behaviour of 15 bees was analysed.

### Pyrethroids and nectar toxins

Six Na^+^ channel activators were used in these experiments (Table 2). The pesticides, cyfluthrin, permethrin, tau-fluvalinate and allethrin I, and the nectar toxin, aconitine, were obtained from Sigma Aldrich (≥99% purity). We administered a dose of 1 ng/μl of each pyrethroid and for aconitine (10 μl of solution = 10ng/bee = 1 ppm). Grayanotoxin I (GTX) was isolated from a methanol extract of dried flowers of *Rhododendron ponticum* using semi-prep high performance liquid chromatograph as reported previously [26]. The concentration of GTX in the nectar of this population of *R*. *ponticum* found on the Isle of Cumrae, Millport Scotland, was determined to be 3.7 ng/μl using methods described in [26]. Therefore, we chose to administer GTX at a dose of 37 ng/bee (10 μl of solution/bee). Pyrethroids and nectar toxins (with the exception of GTX) were dissolved in dimethyl sulphoxide (DMSO) to make stock solutions at a DMSO concentration of 1/1000. A dried sample of GTX was dissolved in water to make a working concentration of 3.7 ppm. Both GTX and control sucrose solutions had DMSO added to them to ensure that the dose of DMSO was consistent between treatments. Stock solutions of cyfluthrin, permethrin, tau-fluvalinate, allethrin I and aconitine were diluted with 1 M sucrose solution to give a working sub-lethal concentration of 1 ppm, equivalent to a dose of 10 ng/μl (except for GTX).

**Table 2:** Compounds used in this Study, the LD_50_ Values for Bees and Their Interactions with Voltage-Gated Sodium Channels.

Compound and Origin	LD_50_ (ppm) in bees^2^	Interaction with Voltage-Gated Sodium Channel
**Grayanotoxin:** Found in members of the *Ericaceae* family, mainly *Rhododendron* spp. [43]	Unknown	Binds to receptor site 2, segment 6, domain IV. Binds preferentially to activated NaV channels and prevents inactivation resulting in repetitive discharges. [43]
**Aconitine:** Found in *Aconitum* spp. plants of the *Ranunculaceae* family, also known as monkshood and wolfsbane. [43]	Unknown	Binds to receptor site 2, segment 6, domain IV. Binds preferentially to activated NaV channels and prevents inactivation resulting in repetitive discharges. [43]
**Pyrethrin:** Found in *Tanacetum* spp. of the Asteraceae family. [5]	1.48	Binding site unknown. [43]
**Allethrin:** A type I pyrethroid. Synthetic homologue of natural cinerin I, of *Tanacetum* spp. The first synthetic pyrethroid. [43]	48.8	Binding site unknown, but may target ‘site 7.’ Inhibits deactivation of NaV channels, prolonging opening. [19, 43]
**Cyfluthrin:** A type I pyrethroid. Synthetic homologue of natural cinerin I, of *Tanacetum* spp. [43]	0.22	Binding site unknown, but may target ‘site 7.’ Inhibits deactivation of NaV channels, prolonging opening. [19]
**Permethrin:** A type I pyrethroid. Synthetic homologue of natural cinerin I, of *Tanacetum* spp. [5]	1.12	Binding site unknown, but may target ‘site 7.’ Inhibits deactivation of NaV channels, prolonging opening. [19]
**Tau-fluvalinate:** A type II pyrethroid (no cyclopropane ring). Synthetic homologue of natural cinerin I, of *Tanacetum* spp. [5]	15.86	Binding site unknown, but may target ‘site 7.’ Inhibits deactivation of NaV channels, prolonging opening. [19]

### Grayanotoxin dose dependent study

Two concentrations of GTX were used for this study: 10 μM (3.7 ppm) and 100 μM (37 ppm) (corresponding to doses of 37 ng/bee and 370 ng/bee, respectively). These concentrations were chosen because they represent the range of concentrations found in nectar [26]. A 1 M sucrose control solution was also used, to which DMSO was added, to result in a DMSO concentration of 1/1000.

### Behavioural Observations

Behavioural observations were manually entered by the experimenter into Observer 5.0 software from Noldus Information Technology B.V. as previously described [36]. The observation period was 10 min per individual. All treatments were observed in a given day of the experiment. The order in which the bees assigned to each treatment were observed was randomized each day. Behaviours recorded are described in Table 3. The behaviours that we chose to record were determined in a pilot study to be the most prevalently observed behaviours; these behaviours have also been observed in previous studies from our laboratory [15, 36]. Three behaviours (walking, still, upside down) were mutually exclusive. A bee was defined as being upside down when it had fallen onto its back and failed to right itself. Wing fanning behaviour and grooming were classified as forms of ‘still’ behaviour (and are represented within this category in the figures). For example, a bee could be both ‘still’ and ‘wing-fanning.’ For this reason, wing fanning and grooming behaviour were analysed separately from the walking, still, and upside down behaviours. All behaviour was continuously recorded over the 10 min interval.

**Table 3:** Behaviours. Descriptions of behaviours recorded using Noldus Observer 5.0, over the ten min observation period.

Behaviour	Description of the Behaviour
Walking	Bee is walking around including on the sides and top of the petri dish
Remaining Still	Bee is standing still, but may be carrying out another behaviour e.g. grooming.
Falling Upside Down	Bee is lying on its back
Abdomen Grooming	Bee is using a leg to groom its abdomen
Leg Grooming	Bee is using one leg to groom another leg
Face Grooming	Bee is using a leg to groom its face
Antennae Grooming	Bee is using a leg to groom its antennae
Proboscis Grooming	Bee is using a leg to groom its proboscis
Wing Fanning	Bee is fluttering its wings or attempting to fly

### Statistics

Generalized linear models (GLM) were used to analyse the behaviour data in IBM SPSS 19.0. A Tweedie model with a log link was used to analyse both percent of the interval and mean duration data. A negative binomial with log link was used to analyse the bout data. Sidak’s pairwise *post hoc* comparisons (PC) were used to determine which treatments had effects that were significantly different from controls.

## Results

### Sodium channel activators and their effect on motor function

**Fig 2:**
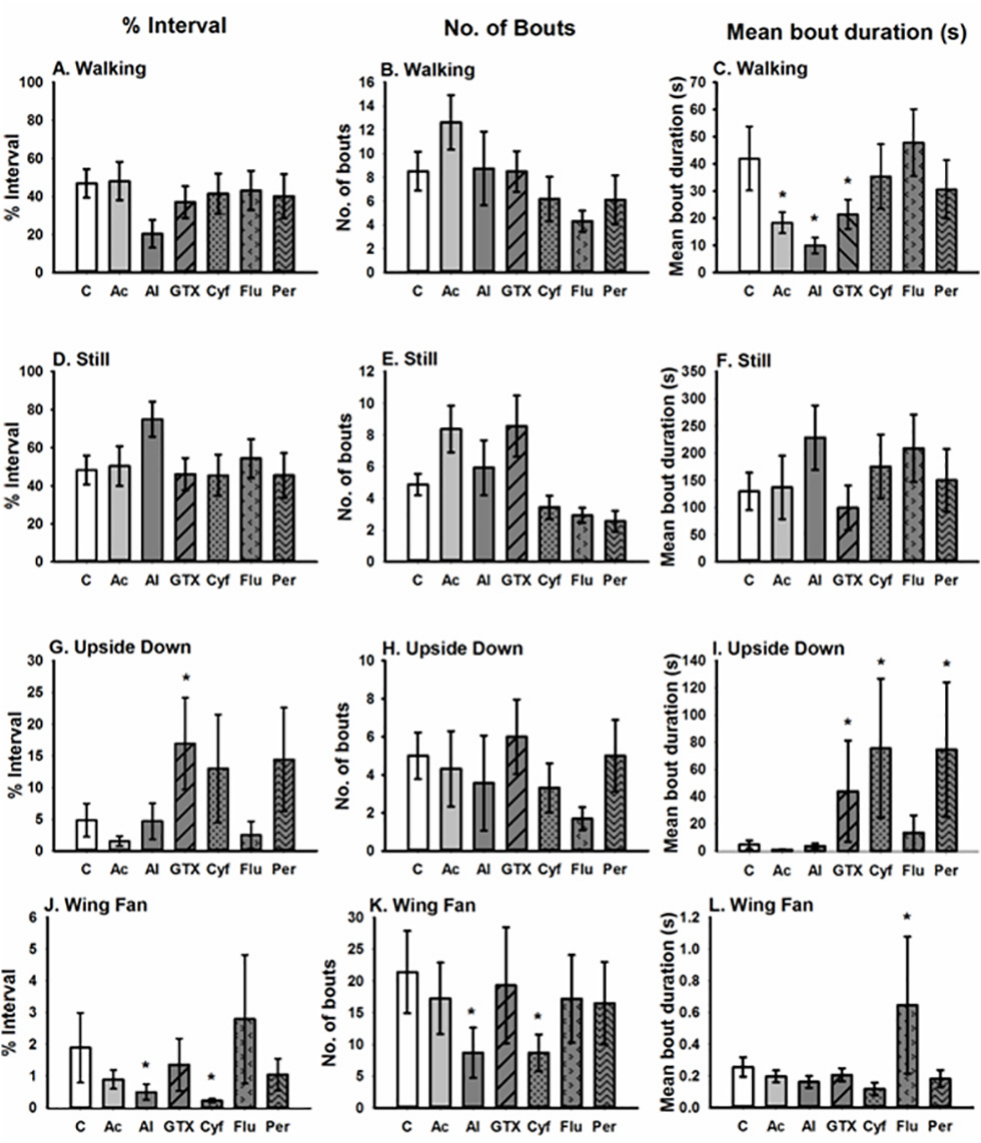
Acute effects of pyrethroids and nectar toxins, at a dose of 10 ng/bee (37 ng/bee for GTX I), on honeybee motor function and wing fanning behaviour. This figure illustrates how compounds affect the percentage of time, number of bouts and mean duration of: (A-C) walking; (D-F) still; (G-I) upside down; (J-L) wing fanning. Sample size N = 16/treatment. * indicates P<0.05. [C = control, Ac = aconitine, Al = allethrin, GTX = grayanotoxin I, Cyf = cyfluthrin, Flu = tau-fluvalinate, Per = permethrin].

**Fig 3:**
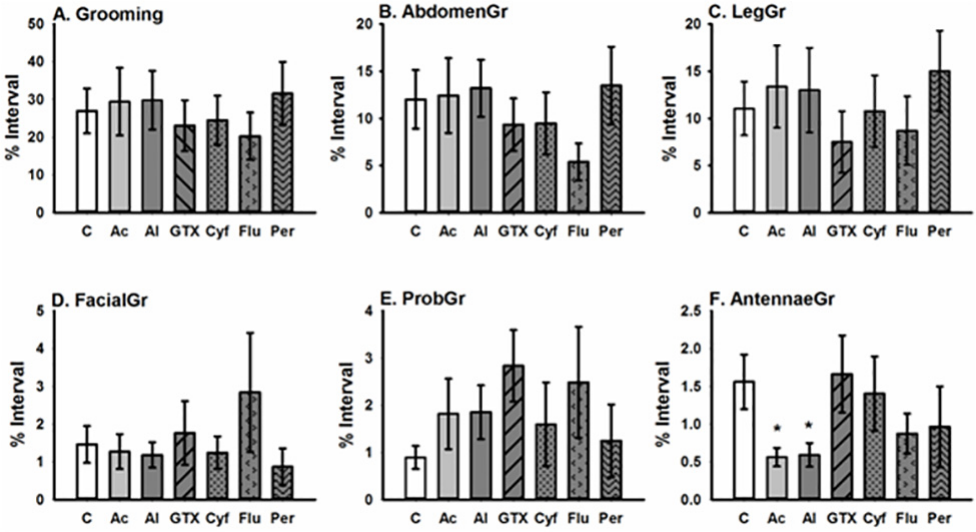
Acute effects of pyrethroids and nectar toxins, at a dose of 10 ng/bee (37 ng/bee for grayanotoxin I), on honeybee grooming behaviour. This figure illustrates how compounds affected the percentage of time that bees spent: (A) grooming (pooled total of all grooming behaviours); (B) abdomen grooming; (C) leg grooming; (D) facial grooming (E) proboscis grooming; (F) antennal grooming. Sample size N = 16/treatment. * indicates P<0.05. [C = control, Ac = aconitine, Al = allethrin, GTX = grayanotoxin I, Cyf = cyfluthrin, Flu = tau-fluvalinate, Per = permethrin].

Control bees spent on average ~50% of their time walking (Fig 2A–2C). These bees also spent an average of ~50% of their time standing still (Fig 2D–2F). Control bees spent only ~5% of their time upside down (Fig 2G–2I). When control bees were standing still, they spent less than 2% of their time wing fanning (Fig 2J–2L) and an average of ~27% of their time grooming (Fig 3).

Bees fed with pyrethroids and nectar toxins that activate sodium channels did not spend less time walking or still than the control group even though bees fed with allethrin, GTX, and aconitine had shorter bouts of walking behaviour than the control group (Fig 2C, Table 4). However, when bees were fed with the pyrethroids and nectar toxins, they spent 5–10% more time upside down (Fig 2G–2I). In particular, bees fed GTX, cyfluthrin and permethrin had significantly longer lasting bouts of upside down behaviour (Fig 2I; Table 4). The only other behaviour affected by pyrethroids and nectar toxins was wing fanning behaviour. Bees fed allethrin and cyfluthrin spent less time wing fanning than the control bees (Fig 2J; Table 4); these bees also had fewer bouts of wing fanning (Fig 2K; Table 4). Tau-fluvalinate, on the other hand, caused bees to have longer bouts of wing fanning compared to the control (Fig 2L; Table 4).

**Table 4:** Pyrethroid/Nectar Toxin Study Summary Statistics. P values and Chi-squared values (with degrees of freedom indicated) for statistical analysis of motor, wing fanning and total grooming behaviour.

	% Interval		Bouts		Mean Duration	
Behaviours	χ_6_^2^	P	χ_6_^2^	P	χ_6_^2^	P
Walking	5.29	0.507	10.0	0.123	18.2	0.006
Still	4.48	0.613	18.6	0.005	7.92	0.244
Upside Down	28.9	<0.001	12.3	0.055	76.0	<0.001
Total Grooming	2.26	0.894	4.67	0.587	4.25	0.642
Wing Fanning	26.7	<0.001	13.9	0.030	19.8	0.003

The amount of time bees spent grooming was unaffected by the pyrethroids and the nectar toxins in our study (Fig 3, S1 Fig, Table 5). The only class of grooming behaviour significantly affected by the compounds we tested was antennal grooming (Fig 3F; Table 5); bees fed with aconitine and allethrin spent less time grooming the antennae than the control bees.

**Table 5:** Pyrethroid/Nectar Toxin Study Summary Statistics. P value and Chi-square values (with degrees of freedom indicated) for statistical analysis of grooming behaviour.

	% Interval		Bouts		Mean Duration	
Behaviours	χ_6_^2^	P	χ_6_^2^	P	χ_6_^2^	P
Proboscis Grooming	6.90	0.330	9.41	0.152	7.87	0.248
Abdomen Grooming	3.97	0.681	10.2	0.116	4.81	0.569
Leg Grooming	2.57	0.860	3.94	0.685	6.41	0.379
Antennae Grooming	15.7	0.015	10.2	0.117	13.9	0.031
Facial Grooming	6.75	0.345	3.30	0.770	8.12	0.229

### Grayanotoxins have dose-dependent effects on honeybee motor function

**Fig 4:**
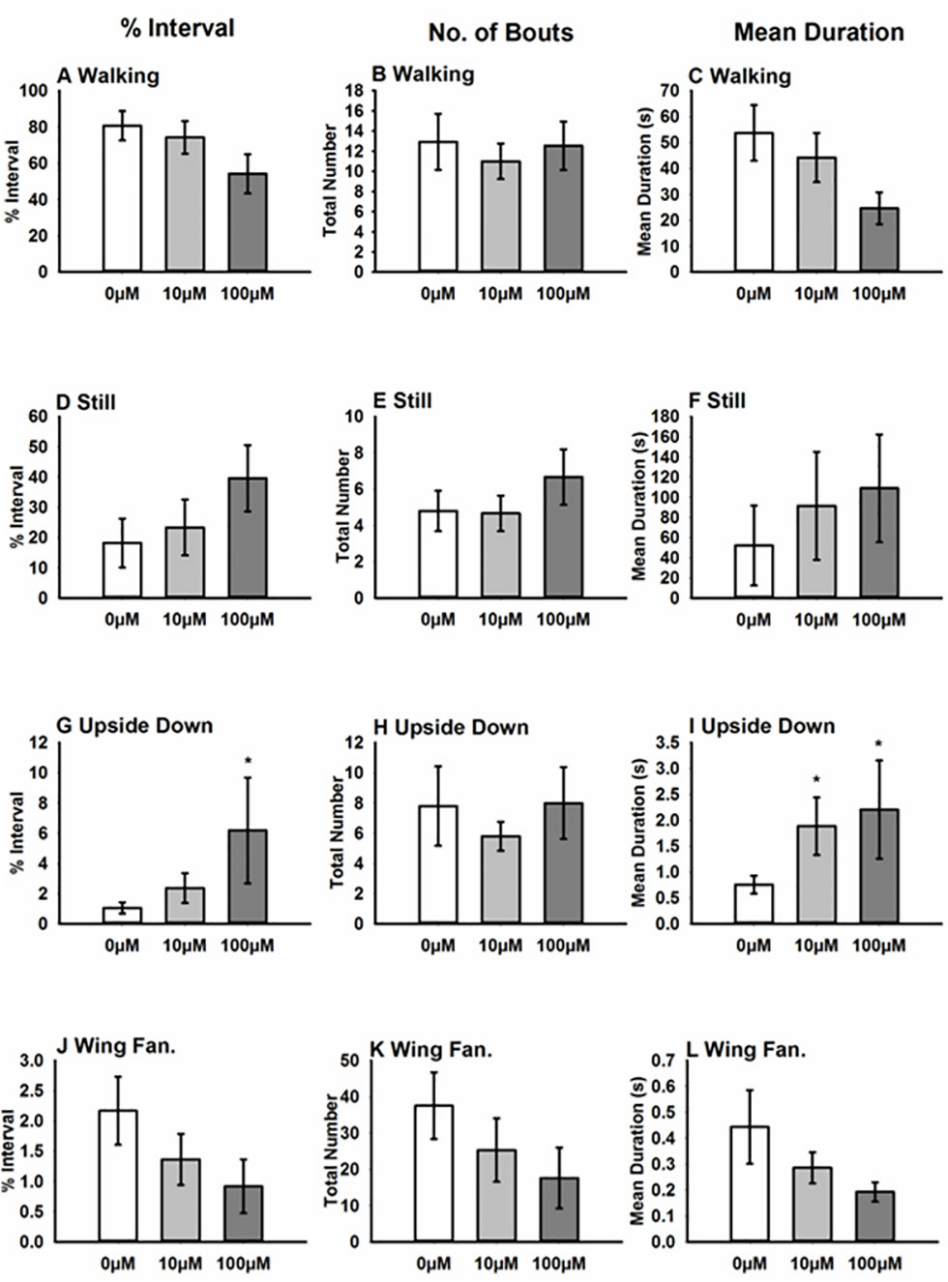
Acute effects of two concentrations of GTX (10 μM and 100 μM) on honeybee motor function and wing fanning. This figure illustrates how the different concentrations of GTX affect the percentage of time, number of bouts and mean duration of: (A-C) walking; (D-F) still; (G-I) upside down; (J-L) wing fanning. Sample size N = 16/treatment. * indicates P<0.05. [C = control, Ac = aconitine, Al = allethrin, GTX = grayanotoxin I, Cyf = cyfluthrin, Flu = tau-fluvalinate, Per = permethrin].

The first experiment tested only one dose of each of the compounds. To establish that the effect we observed was dose-dependent, we tested a concentration series of GTX. GTX was chosen because it had the strongest effect on the ability of bees to perform the righting reflex, resulting in more time spent upside down in the first experiment (Fig 2G). In general, as dose increased, we saw that bees spent more time upside down than control bees (Fig 4; Table 6). We did not find significant effects of dose on any of the other main motor function variables we measured (walking, still, grooming, wing fanning, Fig 4, Table 6). We also examined grooming behaviour in detail (Table 6, S2 Fig); GTX affected the time spent grooming the antennae but it also caused longer bouts of proboscis and facial grooming (S2 Fig and Table 6).

**Table 6:** GTX Dose-Dependent Summary Statistics.

	% Interval		Bouts		Mean Duration	
Behaviours	χ_6_^2^	P	χ_6_^2^	P	χ_6_^2^	P
Walking	2.00	0.368	0.204	0.903	5.58	0.062
Still	4.85	0.090	1.01	0.604	3.11	0.211
Upside Down	15.7	<0.001	0.829	0.661	6.93	0.031
Total Grooming	3.99	0.136	4.09	0.129	1.02	0.601
Abdomen Grooming	1.29	0.526	2.96	0.227	0.981	0.612
Leg Grooming	0.826	0.662	4.17	0.124	0.495	0.781
Facial Grooming	5.07	0.079	7.49	0.024	3.95	0.139
Proboscis Grooming	4.66	0.097	7.07	0.029	1.31	0.519
Antennae Grooming	26.2	<0.001	3.12	0.210	5.37	0.068
Wing Fanning	4.22	0.122	4.13	0.127	4.92	0.085

## Discussion

This study revealed that, on average, low doses of pyrethroids and nectar toxins that target sodium channels mainly affect the ability of honeybees to gain postural control after falling over. The compounds that had the strongest effect on the righting reflex were GTX, cyfluthrin, and permethrin. We also observed that the effect of GTX on the righting reflex was dose-dependent. Two of these compounds, allethrin and cyfluthrin, also reduced wing fanning behaviour, but tau-fluvalinate caused bees to perform bouts of wing fanning that were significantly longer than the control bees. Notably, pyrethroids or nectar toxins that target sodium channels did not have strong effects on grooming behaviour.

We used a suite of compounds from both natural and synthetic sources to test general effects of sodium channel activators on bee behaviour. We expected that the main difference in the compounds that we used was the way their chemical structure interacted with sodium channels to influence behaviour. It is important to emphasize that the structures of the honeybee’s voltage gated sodium channels have not been elucidated–the genes for the subunits of these receptors have not been cloned nor heterologously expressed [44]. Therefore, interpretation of how sodium channel activators affect the honeybee nervous system is based on studies in rats, mosquitos, mites and ticks [44–46]. In these organisms, the target sites are only known for a few of the compounds we tested. For example, GTX and aconitine both target receptor site 2 within the S6 segment of insect voltage-gated sodium channels [47], but the exact binding sites of allethrin, cyfluthrin, permethrin and tau-fluvalinate are unknown. Furthermore, it has been difficult to identify the binding sites of pyrethroids because of their high hydrophobicity and high binding affinity [43, 48]. One review claims that studies of pyrethroid binding to sodium channels in rat brain neurons identified a new receptor site termed ‘site 7’ [19]. However, this site has never been formally described [45]. Interestingly, in general, the nectar toxins aconitine and GTX had a greater influence on honeybee behaviour than the synthetic pesticides. The effect of GTX could be due to the fact that we used a larger dose (3.7 ppm as opposed to 1 ppm of the other compounds), as our experiment that varied the dose of GTX revealed the impact on the righting reflex was dose-dependent. It could also be a result of the complex structure of GTX (Table 2). Futures studies of the structure of honeybee sodium channels will permit a greater understanding of the way that pyrethroids and other toxins interact with these proteins.

From an ecological perspective, it is surprising to find toxins in nectar that activate sodium channels. Plants produce floral nectar as a reward for visiting pollinators. Nectar toxins are metabolically expensive for plants to produce and mainly act as a chemical defence against herbivores. The ecological reasons for this are not clear, but it is possible that nectar containing such compounds could be a mechanism for specialization in plant-pollinator interactions if insect visitors that were not effective pollinators were susceptible to them [24, 26].

Several of the sodium channel activators used in this study affected wing fanning behaviour. Only a few studies of motor function that have used this assay have seen elevated wing behaviours in bees in response to chemical exposure [15, 36, 37]. Within the colony, honeybees stand at the entrance of the hive and rapidly fan their wings, to send air currents through the hive for ventilation and to spread pheromone signals [49]. Fanning is also performed to maintain the temperature, humidity and carbon dioxide levels within the hive and also to concentrate honey [49, 50]. Our data suggest that compounds that target sodium channels affect the circuits governing this behaviour, perhaps indicating that neurons in these circuits express sodium channels composed of different subunits to those in other neurons. Thus, it is possible that when bees are exposed to sodium channel activators (e.g. tau-fluvalinate to treat mites) this affects wing fanning which in turn affects the temperature, humidity and carbon dioxide levels within the hive, which, in turn, may affect brood growth.

Previous studies have shown that bees fed with toxins exhibit changes in behaviour that include failure to perform the righting reflex and more grooming and standing still [51]. The pyrethroids and nectar toxins we assayed affected the righting reflex, but with the exception of GTX, they did not increase the amount of time spent grooming. In fact in the case of aconitine and allethrin, the time spent antennal grooming was lower than the control group. In contrast, the bees given the highest dose of GTX spent more time antennal grooming, and had longer bouts of proboscis and facial grooming. Furthermore, the previous study of toxin-induced ‘malaise’ also reported the occurrence of specific behaviours, such as time spent curled up and abdomen dragging, which we did not observe in this study [51]. Therefore, with the exception of GTX, we predict that ingestion of pyrethroids and aconitine at a 1ppm dose does not cause a malaise reaction in bees. Instead, the influence of these compounds could simply be a disruption of motor function by specifically affecting the neural circuits involved in performing this behaviour.

## Supporting Information

S1 Fig The mean number of bouts and bout durations for the grooming behaviours depicted in Fig 3. (TIF)

S2 Fig The grooming behaviour of the bees fed with doses of GTX in Fig 4. (TIF)

## References

[1] Klein A-M, Vaissiere BE, Cane JH, Steffan-Dewenter I, Cunningham SA, Kremen C, et al Importance of pollinators in changing landscapes for world crops. Proc Biol Sci. 2007; 274(1608):303–13. Epub 2007/02/07. 10.1098/rspb.2006.3721 . 17164193 PMC1702377

[2] Breeze TD, Bailey AP, Balcombe KG, Potts SG. Pollination services in the UK: How important are honeybees? Agriculture, Ecosystems & Environment. 2011; 142(3):137–43. Epub 2011/03/28. 10.1016/j.agee.2011.03.020

[3] Hardstone MC, Scott JG. Is *Apis mellifera* more sensitive to insecticides than other insects? Pest Manag Sci. 2010; 66(11):1171–80. Epub 2010/07/29. 10.1002/ps.2001 . 20672339

[4] Front Matter. Status of Pollinators in North America. Washington D.C.: Natl Academy Pr 2007.

[5] Mullin CA, Frazier M, Frazier JL, Ashcraft S, Simonds R, vanEngelsdorp D, et al High levels of miticides and agrochemicals in North American apiaries: implications for honey bee health. PLoS One. 2010; 5(3):e9754 Epub 2010/03/19. 10.1371/journal.pone.0009754 . 20333298 PMC2841636

[6] vanEngelsdorp D, Evans JD, Saegerman C, Mullin C, Haubruge E, Nguyen BK, et al Colony Collapse Disorder: A Descriptive Study. PLoS One. 2009; 4(8):e6481 Epub 2009/08/03. 10.1371/journal.pone.0006481 . 19649264 PMC2715894

[7] Baron GL, Raine NE, Brown MJF. Impact of chronic exposure to a pyrethroid pesticide on bumblebees and interactions with a trypanosome parasite. J Appl Ecol. 2014; 51(2):460–9. Epub 2014/01/19. 10.1111/1365-2664.12205

[8] Becher MA, Osborne JL, Thorbek P, Kennedy PJ, Grimm V. Review: towards a systems approach for understanding honeybee decline: a stocktaking and synthesis of existing models. J Appl Ecol. 2013; 50(4):868–80. Epub 2013/06/10. 10.1111/1365-2664.12112 . 24223431 PMC3810709

[9] Retschnig G, Neumann P, Williams GR. Thiacloprid–*Nosema ceranae* interactions in honey bees: Host survivorship but not parasite reproduction is dependent on pesticide dose. J Invertebr Pathol. 2014; 118:18–9. Epub 2014/03/01. 10.1016/j.jip.2014.02.008 . 24594300

[10] Goulson D. Review: An overview of the environmental risks posed by neonicotinoid insecticides. J Appl Ecol. 2013; 50(4):977–87. Epub 2013/06/13. 10.1111/1365-2664.12111

[11] Bortolotti L, Montanari R, Marcelino J, Medrzycki P, Maini S, Porrini C. Effects of sub-lethal imidacloprid doses on the homing rate and foraging activity of honey bees. Bulletin of Insectology. 2003; 56:63–8.

[12] Ramirez-Romero R, Chaufaux J, Pham-Delegue M. Effects of Cry1Ab protoxin, deltamethrin and imidacloprid on the foraging activity and the learning performances of the honeybee *Apis mellifera*, a comparative approach. Apidologie. 2005; 36(4):601 Epub 2005/11/15. 10.1051/apido:2005039

[13] Yang EC, Chuang YC, Chen YL, Chang LH. Abnormal foraging behavior induced by sublethal dosage of imidacloprid in the honey bee (Hymenoptera: Apidae). J Econ Entomol. 2008; 101(6):1743–8. Epub 2008/12/01. doi: 10.1603/0022-0493-101.6.1743 . 19133451

[14] Williamson SM, Wright GA. Exposure to multiple cholinergic pesticides impairs olfactory learning and memory in honeybees. J Exp Biol. 2013; 216(10):1799–807. Epub 2013/02/07. 10.1242/jeb.083931 . 23393272 PMC3641805

[15] Williamson SM, Willis SJ, Wright GA. Exposure to neonicotinoids influences the motor function of adult worker honeybees. Ecotoxicology. 2014; 23(8):1409–18. Epub 2014/07/11. 10.1007/s10646-014-1283-x . 25011924 PMC4165879

[16] Medrzycki P, Montanari R, Bortolotti L, Sabatini AG, Maini S, Porrini C. Effects of imidacloprid administered in sub-lethal doses on honey bee behaviour. Laboratory tests. Bulletin of Insectology. 2003; 56:59–62.

[17] Department for Environment, Food & Rural Affairs (DEFRA). Neonicotinoids. 2013. Available:https://www.gov.uk/government/publications/neonicotinoids

[18] Katsuda Y. Progress and future of pyrethroids. Top Curr Chem. 2012; 314:1–30. Epub 2011/11/03. 10.1007/128_2011_252 . 22048685

[19] Wang S-Y, Wang GK. Voltage-gated sodium channels as primary targets of diverse lipid-soluble neurotoxins. Cell Signal. 2003; 15(2):151–9. Epub 2002/10/12. 10.1016/S0898-6568(02)00085-2 . 12464386

[20] Zhou T, Zhou W, Wang Q, Dai P-L, Liu F, Zhang Y-L, et al Effects of pyrethroids on neuronal excitability of adult honeybees *Apis mellifera* . Pest Biochem Physiol. 2011; 100(1):35–40. Epub 2011/02/23. 10.1016/j.pestbp.2011.02.001

[21] ChemSpider. cis-Cyfluthrin. R Soc Chem. 2015; Available from: http://www.chemspider.com/Chemical-Structure.45482.html.

[22] ChemSpider. Permethrin. R Soc Chem. 2015; Available: http://www.chemspider.com/Chemical-Structure.36845.html?rid=2445d37c-1139-48de-8bbf-67cfb0233d2a.

[23] ChemSpider. Tau-Fluvalinate. R Soc Chem. 2015; Available: http://www.chemspider.com/Chemical-Structure.82865.html?rid=b88d4ffb-17c0-4ab7-975b-86c7e54f6429.

[24] Adler LS. The ecological significance of toxic nectar. Oikos. 2000:409–20. Epub 2000/08/08. 10.1034/j.1600-0706.2000.910301.x

[25] Gosselin M, Michez D, Vanderplanck M, Roelants D, Glauser G, Rasmont P. Does *Aconitum septentrionale* chemically protect floral rewards to the advantage of specialist bumblebees? Ecol Entomol. 2013; 38(4):400–7. Epub 2013/05/20. 10.1111/een.12032

[26] Tiedeken EJ, Stout JC, Stevenson PC, Wright GA. Bumblebees are not deterred by ecologically relevant concentrations of nectar toxins. J Exp Biol. 2014; 217(9):1620–5. Epub 2014/02/13. 10.1242/jeb.097543 . 24526720 PMC4006588

[27] Grdiša M, Babić S, Periša M, Carović‐Stanko K, Kolak I, Liber Z, et al Chemical diversity of the natural populations of Dalmatian Pyrethrum (*Tanacetum cinerariifolium* (TREVIR.) SCH. BIP.) in Croatia. Chem Biodivers. 2013; 10(3):460–72. Epub 2013/03/13. 10.1002/cbdv.201200015 . 23495162

[28] Choudhary A, Sharma DC. Dynamics of pesticide residues in nectar and pollen of mustard (*Brassica juncea* (L.) Czern.) grown in Himachal Pradesh (India). Environ Monit Assess. 2008; 144(1–3):143–50. Epub 2007/10/19. 10.1007/s10661-007-9952-3 . 17952621

[29] Shires SW, Blanc JL, Debray P, Forbes S, Louveaux J. Field experiments on the effects of a new pyrethroid insecticide WL‐85871 on bees foraging artificial aphid honeydew on winter wheat. Pestic Sci. 1984; 15(6):543–52. Epub 2006/06/26. 10.1002/ps.2780150603

[30] Bishop JA. Bumble bees (*Bombus hypnorum*) collect aphid honeydew on stone pine (*Pinus pumila*) in the Russian Far East. J Kans Entomol Soc. 1994; 67:220–2.

[31] Amad M, Arif MI, Denholm I. High resistance of field populations of the cotton aphid *Aphis gossypii* Glover (Homoptera: Aphididae) to pyrethroid insecticides in Pakistan. J Econ Entomol. 2003; 96(3):875–8. Epub 2003/06/01. doi: 10.1093/jee/96.3.875 . 12852630

[32] Decourtye A, Devillers J, Genecque E, Le Menach K, Budzinski H, Cluzeau S, et al Comparative sublethal toxicity of nine pesticides on olfactory learning performances of the honeybee *Apis mellifera* . Arch Environ Contam Toxicol. 2005; 48(2):242–50. Epub 2005/02/15. 10.1007/s00244-003-0262-7 . 15750780

[33] Frost EH, Shutler D, Hillier NK. Effects of fluvalinate on honey bee learning, memory, responsiveness to sucrose, and survival. J Exp Biol. 2013; 216(15):2931–8. Epub 2013/04/25. 10.1242/jeb.086538 . 23619403

[34] Gunasekara AS. Environmental fate of pyrethrins California Department of Pesticide Regulation 2004; Available:http://cdpr.ca.gov/docs/emon/pubs/fatememo/pyrethrin_efate2.pdf.

[35] Johnson RM, Dahlgren L, Siegfried BD, Ellis MD. Acaricide, fungicide and drug interactions in honey bees (*Apis mellifera*). PloS One. 2013; 8(1):e54092 Epub 2013/01/29. 10.1371/journal.pone.0054092 23382869 PMC3558502

[36] Maze IS, Wright GA, Mustard JA. Acute ethanol ingestion produces dose-dependent effects on motor behavior in the honey bee (*Apis mellifera*). J Inesct Physiol. 2006; 52(11):1243–53. Epub 2006/09/20. 10.1016/j.jinsphys.2006.09.006 . 17070538 PMC1712673

[37] Fussnecker BL, Smith BH, Mustard JA. Octopamine and tyramine influence the behavioral profile of locomotor activity in the honey bee (*Apis mellifera*). J Insect Physiol. 2006; 52(10):1083–92. Epub 2006/09/05. 10.1016/j.jinsphys.2006.07.008 . 17028016 PMC1712669

[38] ChemSpider. Grayanotoxin. R Soc Chem. 2015; Available: http://www.chemspider.com/Chemical-Structure.23339126.html?rid=a9b411ec-d454-433d-a3da-13e7b7d1ec3b.

[39] ChemSpider. Aconitine. R Soc Chem. 2015; Available: http://www.chemspider.com/Chemical-Structure.214292.html.

[40] ChemSpider. Chrysanthemumic acid ester of (±)-allethrolone. R Soc Chem. 2015; Available: http://www.chemspider.com/Chemical-Structure.10958.html.

[41] ChemSpider. 3-Allyl-2-methyl-4-oxo-2-cyclopenten-1-yl-3-[(2E)-2-buten-2-yl]-2,2-dimethylcyclopropaneca. R Soc Chem. 2015; Available: http://www.chemspider.com/Chemical-Structure.13899140.html.

[42] Bitterman ME, Menzel R, Fietz A, Schäfer S. Classical conditioning of proboscis extension in honeybees (*Apis mellifera*). J Comp Phsyiol. 1983; 97(2):107. doi: 10.1037/0735-7036.97.2.107. 6872507

[43] Du Y, Nomura Y, Satar G, Hu Z, Nauen R, He SY, et al Molecular evidence for dual pyrethroid-receptor sites on a mosquito sodium channel. Proc Natl Acad Sci. 2013; 110(29):11785–90. Epub 2013/07/2. 10.1073/pnas.1305118110 . 23821746 PMC3718148

[44] Kadala A, Charreton M, Jakob I, Cens T, Rousset M, Chahine M, et al Pyrethroids differentially alter voltage-gated sodium channels from the honeybee central olfactory neurons. PLoS One. 2014; 9(11)e112194. Epub 2014/11/12. 10.1371/journal.pone.0112194 . 25390654 PMC4229128

[45] O'Reilly AO, Williamson MS, González‐Cabrera J, Turberg A, Field LM, Wallace BA, et al Predictive 3D modelling of the interactions of pyrethroids with the voltage‐gated sodium channels of ticks and mites. Pest Manag Sci. 2014; 70(3):369–77. Epub 2013/08/01. 10.1002/ps.3561 . 23589444

[46] Lombet A, Mourre C, Lazdunski M. Interaction of insecticides of the pyrethroid family with specific binding sites on the voltage-dependent sodium channel from mammalian brain. Brain Res. 1988; 459(1):44–53. Epub 1988/08/30. 10.1016/0006-8993(88)90284-3 . 2844361

[47] Catterall WA, Cestèle S, Yarov-Yarovoy V, Frank HY, Konoki K, Scheuer T. Voltage-gated ion channels and gating modifier toxins. Toxicon. 2007; 49(2):124–41. Epub 2006/09/28. 10.1016/j.toxicon.2006.09.022 17239913

[48] Tan J, Liu Z, Wang R, Huang ZY, Chen AC, Gurevitz M, et al Identification of amino acid residues in the insect sodium channel critical for pyrethroid binding. Mol Pharmacol. 2005; 67(2):513–22. Epub 2004/11/03. 10.1124/mol.104.006205 . 15525757

[49] Su S, Albert S, Zhang S, Maier S, Chen S, Du H, et al Non-destructive genotyping and genetic variation of fanning in a honey bee colony. J Insect Physiol. 2007; 53(5):411–7. Epub 2007/02/01. 10.1016/j.jinsphys.2007.01.002 . 17383675

[50] Winston M. The Biology of the Honeybee. USA: Harvard University Press; 1987.

[51] Hurst V, Stevenson P, Wright G. Toxins induce ‘malaise’ behaviour in the honeybee (*Apis mellifera*). J Comp Physiol A. 2014; 200(10):881–90. Epub 2014/08/23. 10.1007/s00359-014-0932-0 . 25149875 PMC4169619

