# Supplementary figures and images for: Pyrethroids and Nectar Toxins Have Subtle Effects on the Motor Function, Grooming and Wing Fanning Behaviour of Honeybees (Apis mellifera)
Source: PLoS One. 2015 Aug 17;10(8):e0133733. doi: 10.1371/journal.pone.0133733 (PMC4539190; doi:10.1371/journal.pone.0133733)

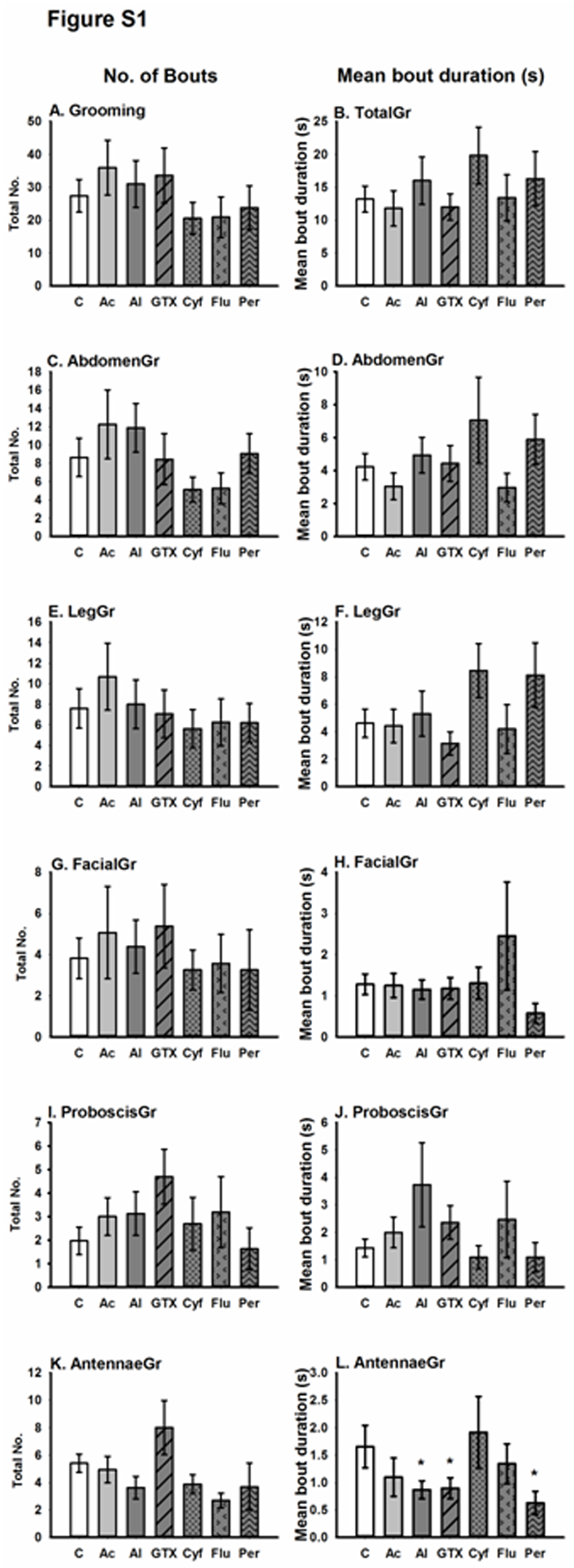

Supplement: S1 Fig — (TIF) [file pone.0133733.s001.tif]

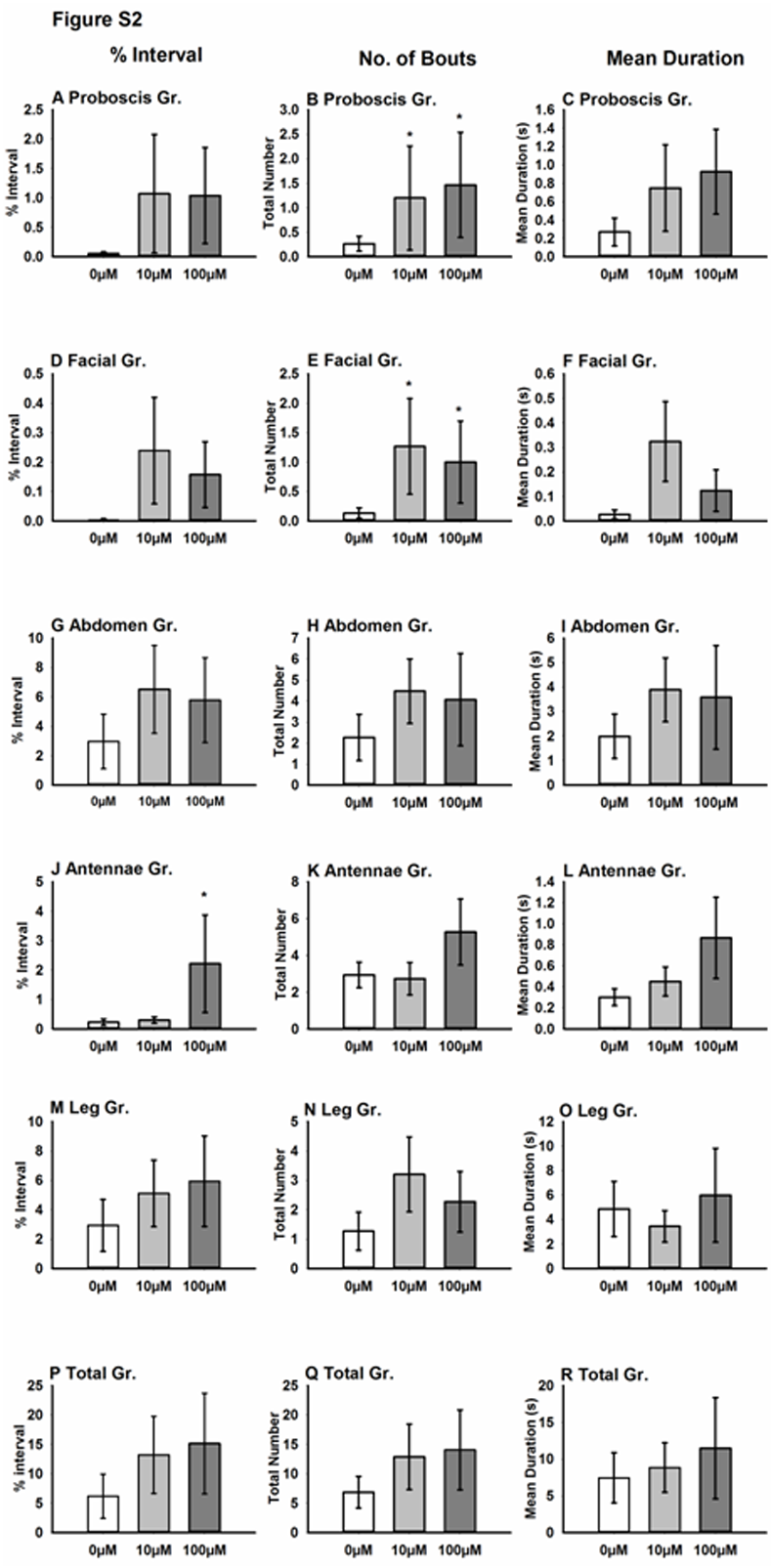

Supplement: S2 Fig — (TIF) [file pone.0133733.s002.tif]
